# Prevalence and antimicrobial susceptibility profile of bacteria isolated from the hands of housemaids in Jimma City, Ethiopia

**DOI:** 10.3389/fpubh.2023.1301685

**Published:** 2024-01-29

**Authors:** Tadele Shiwito Ango, Negalgn Byadgie Gelaw, Girma Mamo Zegene, Tizita Teshome, Tesfalem Getahun

**Affiliations:** ^1^Department of Public Health, Mizan Aman Health Science College, Mizan Aman, Southwest Ethiopia People Regional State, Ethiopia; ^2^Department of Environmental Health Sciences and Technology, Institute of Health, Jimma University, Jimma, Ethiopia

**Keywords:** bacterial isolate, antimicrobial resistance, housemaids, Jimma City, Ethiopia

## Abstract

**Introduction:**

Bacterial pathogens continue to be a major cause of foodborne gastroenteritis in humans and remain a public health problem. Housemaids operating inside a kitchen could be the source of infection and may transmit disease-inflicting pathogens through contaminated hands.

**Objective:**

This study aimed to assess the prevalence and antimicrobial susceptibility profile of bacteria isolated from the hands of housemaids in Jimma City, Ethiopia.

**Methods:**

A laboratory-based cross-sectional study was employed among 234 housemaids. Hand swab samples from the dominant hand of the study participants were collected under sterile conditions following standard operating procedures. Then, in the laboratory, the swabs were inoculated aseptically using streak-plating methods on the growth media, such as mannitol salt agar [*Staphylococcus aureus* and coagulase-negative staphylococci], MacConkey agar [*Klebsiella* species and *Proteus* species], salmonella-shigella agar [*Salmonella* species and *Shigella* species], and eosin methylene blue agar [*Escherichia coli* (*E. coli*)]. In addition, a set of biochemical tests was applied to examine bacterial species. Data were double-entered into EpiData version 3.1 and then exported to the Statistical Package for Social Science (SPSS) version 26 for further analysis. Descriptive analyses were summarized using frequency and percentage.

**Results:**

The proportion of housemaids’ hands containing one or more positive bacterial isolates was 72% (95% CI: 66.2, 77.8). The dominant bacterial isolates were *Staphylococcus aureus* (31.6%), *Escherichia coli* (21.3%), *Salmonella* species (1.3%), *Shigella* species (6.7%), *Klebsiella* species (23.1%) and *Proteus* species (14.7%). Fingernail status (AOR =15.31, 95% CI: 10.372, 22.595) and the removal of a watch, ring, and bracelet during hand washing (AOR = 20.844, 95% CI: 2.190, 9.842) were significantly associated with the prevalence of bacterial isolation. Most *Staphylococcus aureus* isolates were susceptible to chloramphenicol (98.6%). *Escherichia coli* isolates were susceptible to tetracycline (75%), ceftriaxone (79.2%), chloramphenicol (87.5%), and ceftazidime (77.1%). Eighty percent of isolated *Shigella* species were susceptible to chloramphenicol and gentamicin respectively. In addition, *Klebsiella* and *Proteus species* exhibited high susceptibility to chloramphenicol. However, their isolates showed resistance against a number of the tested antimicrobials. *Staphylococcus aureus* isolates (28.2%) were resistance to tetracycline. Moreover, One-quarter of *Escherichia coli* isolates were resistance to tetracycline, ceftriaxone, chloramphenicol, and ceftazidime. Whereas 46.7% and 48.5% of isolated *Shigella* species and Proteus species were resistance to tetracycline and ceftriaxone.

**Conclusion:**

The hands of housemaids are important potential sources of pathogenic bacteria that would result in the potential risk of foodborne diseases. Most bacteria isolates were resistant to tetracycline, ceftriaxone, and ceftazidime. Therefore, practicing good hand hygiene helps to prevent and control the spread of antimicrobial-resistant microbes.

## 1 Introduction

Enteric bacterial pathogens are a major cause of foodborne gastroenteritis in humans and remain a public health problem worldwide (1). They are common foodborne disease agents and persist as a major public health threat. A study revealed that food commodities were contaminated by food handlers or housemaids (2). Moreover, the majority of foodborne outbreak causative agents enter the body through the ingestion of contaminated food (3, 4). Banik et al. state that foodborne illnesses occurred after the entrance of those disease-causing microbes into the food supply chain (5). In 2020, the World Health Organization reported approximately 600 million cases and 420,000 deaths related to contaminated food around the world (3). According to the Centers for Disease Control and Prevention estimates, approximately 1 in 6 Americans (48 million people) get sick, 128,000 are hospitalized, and 3,000 die of foodborne diseases each year (6). However, the problem is severe in developing countries, including Ethiopia. Furthermore, it was estimated that approximately 700,000 deaths per year in Africa are caused by foodborne diseases (7). The summary report of the Federal Ministry of Health revealed that the annual incidences of foodborne illnesses ranged from 3.4 to 9.3% in Ethiopia (8). In addition, several studies have been performed to assess and estimate pathogenic bacteria and related rates of infection in Ethiopia (1, 7, 9–20).

The fecal–oral route of pathogen transmission is the most common among the other methods of infection transmission for heterogeneous pathogens (21, 22). In this sense, varieties of bacterial isolates, particularly *Staphylococcus aureus*, *Klebsiella* species, *Proteus* species, *E. coli*, *Shigella* species, *Salmonella* species, *Campylobacter*, *Vibrio cholerae*, and *Streptococcus pneumonia*, might be ingested that results from the hand contact with feces (5, 12–14, 17, 22–26). Furthermore, studies revealed that bacterial pathogens were the most extensively identified infectious agent for the majority of foodborne outbreak types (8, 11). Housemaids perform various daily activities and work at home; their hands quickly become contaminated with different kinds of microbes and therefore, become asymptomatic carriers of pathogens.

The public health importance of bacterial infection continues to pose a challenge to community health systems worldwide (27–29). Food poisoning (gastroenteritis), skin, ear, or sinus infections, sexually transmitted infections, bacterial pneumonia, and urinary tract infections are typical bacterial infections (6, 30). For instance, *S. aureus* produces toxins that cause staphylococcal food poisoning and gastroenteritis with emesis and with or without diarrhea (23, 31). Infections associated with *Salmonella* species and *Shigella* species are among the major public health problems in many countries, including Ethiopia (7, 9). For instance, *Salmonella* species is the most common cause of foodborne illnesses (23). *Shigella* causes a foodborne illness with common symptoms of diarrhea, fever, and stomach cramps (7). The estimated annual incidences of *Shigella* species and *Salmonella* species are 165 and 25 million, respectively (9). Moreover, a study disclosed that *Klebsiella* species cause infections at multiple sites in the bodies of people with preexisting health conditions (32).

Due to the high prevalence of antimicrobial-resistant (AMR) pathogens, advances in infection control have not completely eradicated the problem (13). In addition, the constant increase in AMR bacterial strains has become an important clinical problem (33, 34). Those AMR bacterial strains include members of Enterobacteriaceae and continue the increasing concern, which could lead to the narrowing of available therapeutic options (34, 35). Antimicrobial resistance could be determined by many contributing factors. The microbial evolution and transmission of genetic determinants of resistance between microbes enable the spread of pathogenic bacteria (34–38). In addition, the widespread and prolonged use of antibiotics leads to the emergence of resistant bacterial pathogens (13, 18, 38). Moreover, the problems of infectious diseases were worsened by the improper use of antibiotics by humans and animals, which contributed to the rise of AMR globally (13, 33–41). In sum, AMR is an emerging global challenge that results in the spread of infectious diseases that affect human populations (38, 39).

Therefore, pathogenic microbes continue to challenge the healthcare systems in developing countries, including Ethiopia (40). Evidence from studies revealed an increasing incidence of multidrug resistance in foodborne pathogens, particularly to the commonly used antimicrobial agents (40, 41). Most studies in Ethiopia have been conducted in institutions such as hospitals, mass food processing, and catering establishments (11, 13, 14, 18). There is a paucity of data showing profiles of bacteria isolated from the hands of housemaids and their antimicrobial susceptibility profile in dwellings, particularly in the study area. Therefore, the present study aimed to assess the prevalence and antimicrobial susceptibility profile of bacteria isolated from the hands of housemaids in Jimma City, Ethiopia.

## 2 Materials and methods

### 2.1 Study area, design, and period

The current laboratory-based cross-sectional survey was conducted in residential settings in Jimma City, Southwest Ethiopia, from April to June 2022. Jimma City is located 352 km southwest of Addis Ababa. The physical location of Jimma City lies between latitude 7°41′ N and longitude 36°50′ E. It has an average altitude of 1,780 m above sea level. It receives a mean annual rainfall of approximately 1,530 mm. The mean annual minimum and maximum temperatures of Jimma City are 14.4 and 26.7°C, respectively (42).

### 2.2 Source and study population

All housemaids who have been engaged in work in Jimma City were the source population. Those housemaids who could fulfill the eligibility criteria and were available during the data collection period were considered the study population.

### 2.3 Eligibility criteria

Housemaids who reported having respiratory infections such as the common cold or fecal–oral diseases such as diarrhea, as well as those who had pores, skin irritation, inflammation, eczema, or scars on their palms during the data collection period, were not included in the analysis.

### 2.4 Sample size determination and sampling technique

The sample size was determined by applying Yamane’s simplified formula for proportions. A 95% confidence interval (CI) and 0.5 or 50% proportion were assumed


$$n=\frac{N}{1+{Ne}^{2}}$$

$$n=\frac{455}{\;1+455{\left(0.05\right)}^{2}}\;=213$$

where *n* is the sample size, *N* is the population size, and *e* is the level of precision (5%). After considering a 10% sample size (21 study subjects) with a non-response rate, the final sample size was 213 + 21 = 234.

All residential settings in Jimma City were included in the study. An inventory assessment was conducted to gather information about housemaids employed in residential settings, and data about the total number of housemaids were obtained from households and local administrations [Kebele]. In sum, 455 housemaids were engaged in residential settings in Jimma City (43). Those housemaids who were available during the data collection period were included until the sample size (234) was fulfilled. To minimize sampling bias, after convincing the heads of households, their house number was used for identification.

### 2.5 Data collection techniques

Data collection tools for sociodemographic data and other relevant data related to hand hygiene practices among housemaids were adapted from the World Health Organization and published articles (44–46). Whereas, hand swab sample collection procedures followed the guidelines of the Clinical and Laboratory Standards Institute (CLSI), the American Type Culture Collection, and others (47–49).

Data were collected by the data collectors after obtaining written informed consent using a pre-tested semi-structured questionnaire and observation designed to obtain sociodemographic data such as sex, age and educational status, and other relevant data related to housemaids’ hand hygiene practices such as fingernail status, frequent handwashing, handwashing method, use of soap and water for frequent handwashing, following the five steps to washing hands in the right way, removal of watch, ring, and bracelets during handwashing, and time in second to wash hands. The data were collected from the study participants after receiving their written informed consent and after receiving the ethical approval for the study from the Institutional Review Board of the Institute of Health, Jimma University.

Three data collectors with prior experience in the field and who were fluent in speaking and reading in the local language and English were hired. The data collection survey underwent 5-day training sessions on informed consent and data collection procedures from 20 to 25 March 2022. The data collectors were two individuals with Bachelor of Science degrees in medical laboratory technology and one individual with a Bachelor of Science degree in environmental health science.

### 2.6 Laboratory data, analysis, and interpretation

For the laboratory investigation of the commensal microbes from the hands of housemaids, a swab sample was collected following standard operating procedures of CLSI, American-type culture collection, and other guidelines (47–49). In advance, hand swab samples were collected following sterile conditions for the segregation of commensal microbes.

#### 2.6.1 Sample collection and transport

The samples were collected and transported using sterile cotton swabs and 10 mL saline-filled sterile test containers. Following handwashing, the participant’s hands were sampled for the hand swabs by rubbing the entire surface with sterile, moistened cotton-tipped swabs. The sample was then placed or soaked in a labeled 0.85% saline solution containing sterile test tubes for microbial culturing. Nevertheless, no prior notice was given, and extra hand cleanliness was not practiced when collecting samples (18, 50). Three well-trained laboratory personnel gathered swab samples using accepted aseptic methods. Samples were transferred to Jimma University’s Department of Medical Microbiology Laboratory soon after collection. Next, in the laboratory, the samples were enhanced in a nutrient broth for a whole day to promote bacterial recovery, as handwashing has an impact on the survival of the bacteria that were collected.

#### 2.6.2 Sample culturing and identification

The most popular techniques for identifying bacteria include the use of differential media, which makes it simpler to separate colonies of desired microorganisms from other colonies growing on the same plate, or selective media, which can prevent or reduce the growth of undesirable commensal microbes (47, 49). By providing the growth media, it is essential to grow and maintain them in carefully regulated laboratory settings. The preparation of each culture medium utilized in this investigation was performed in accordance with the manufacturer’s instructions, and aseptic culturing techniques were employed. A loop full of each hand swab sample enriched on the nutrient broth in the laboratory soon after collection was inoculated aseptically using streak-plating methods on the selective and differential such as mannitol salt agar (MSA) (*S. aureus* and Coagulase-negative staphylococci), MacConkey agar (MCA) (*Klebsiella* species and *Proteus* species), salmonella-shigella agar (SSA) (*Salmonella* species and *Shigella* species), and eosin methylene blue agar (EMBA) (*E. coli*). Then, it was incubated at 37°C for 24 h. Following an incubation period, the culture plates were inspected to see whether there was any suspected bacterial growth present (positive) or absent (negative). Biochemical and morphological testing were used to corroborate the positive laboratory results.

#### 2.6.3 Biochemical tests

**Figure 1:**
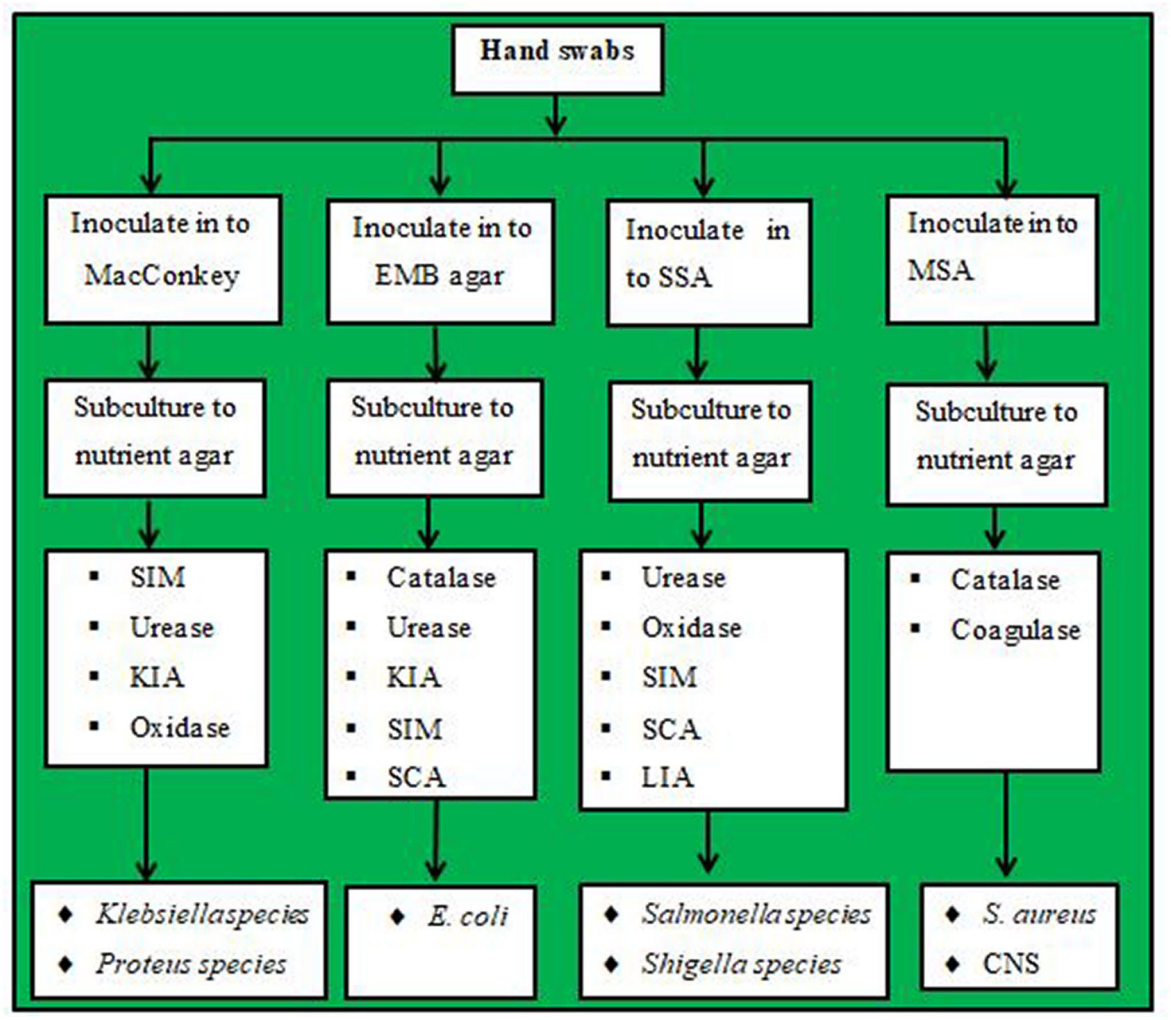
Laboratory flowchart showing bacteria isolations from hand swab samples.

The single colony of bacteria grown on selective and differential media was then subcultured into nutrient agar to determine growth patterns and for further biochemical tests. Then, after obtaining pure colonies, identification of bacteria isolates was performed by using standard microbiology techniques such as the morphology of its colonies and a battery (set) of biochemical tests such as a response on catalase, coagulase, oxidase, Simon citrate agar (SCA), urease, sulfide indole motility (SIM), Kliger’s Iron Agar (KIA), and gas and hydrogen sulfide (H_2_S) generation (17). The isolation and identification of bacteria from the hand swabs from the hands of housemaids are shown (Figure 1).

#### 2.6.4 Antimicrobial susceptibility tests

Antimicrobial susceptibility tests were performed on Muller Hinton Agar (HIMEDIA, TITAN BIOTECH LTD, Rajasthan, India) by the disk diffusion method. The following antimicrobial drugs were used to test susceptibility: tetracycline (30 μg), ceftriaxone (30 μg), chloramphenicol (30 μg), gentamicin (10 μg), and ceftazidime (30 μg). The selection of drugs was based on availability and pieces of literature (17, 48). The selections of drugs for antimicrobial sensitivity tests were based on the standards for antimicrobial susceptibility guidelines (48), pieces of literature (13, 14, 18), and the current availability of drugs in the market that the community is accessing. The sensitivity, intermediate, and resistance of the bacterial isolates were interpreted according to CLSI guidelines (48).

All culture media used for antimicrobial susceptibility tests were prepared according to the manufacturer’s instructions, and culturing procedures were carried out aseptically. Each batch of the prepared media was checked for sterility by incubating the sample medium at 37°C for a day (14). *Staphylococcus aureus* ATCC25923 and *E. coli* ATCC25922, sensitive to all antimicrobial agents, were used as control strains (48). The zone diameter interpretive standards for the determination of antimicrobial agents are shown below (Table 1).

**Table 1:** Zone diameter interpretive standards for the determination of antimicrobial agent sensitivity and resistance tested by disk diffusion method.

Bacteria category	Antimicrobial agent	Disk content	Interpretive categories and zone diameter breakpoints nearest whole mm		
			Sensitive	Intermediate	Resistant
Gram-positive bacteria [*S. aureus* and Coagulase-negative staphylococci]	Tetracycline	30 μg	≥19	15–18	≤14
	Chloramphenicol	30 μg	≥18	13–17	≤12
	Gentamicin	10 μg	≥15	13–14	≤12
Enterobacteriaceae [*Escherichia coli, Salmonella* species*, Shigella* species*, Klebsiella* species, *and Proteus* species]	Tetracycline	30 μg	≥15	12–14	≤11
	Ceftriaxone	30 μg	≥23	20–22	≤19
	Chloramphenicol	30 μg	≥18	13–17	≤12
	Gentamicin	10 μg	≥15	13–14	≤12
	Ceftazidime	30 μg	≥21	18–20	≤17

### 2.7 Quality control

The data collection questionnaire was designed, modified, and contextualized after reviewing related pieces of literature (44–46). Before data collection, a pre-test was conducted among 10% of the total sample size of the study subjects in Mizan-Aman town to determine whether any corrections were made or not.

To manage the quality of work, standard operating procedures have been strictly adhered to in laboratory tests for the investigation of commensal microbes (49). In addition, the proper functioning of the instruments utilized was checked before processing samples, and the known strains of selected organisms (*S. aureus* ATCC25923 and *E. coli* ATCC25922) were used for comparison purposes while distinguishing quality. The hand swab samples were collected aseptically, and the temperature range until the final laboratory analysis was checked. Then, the bacterial enrichment broth and growth media sterility were ensured using an autoclave and sample media storage in an incubator overnight, respectively. Interpretation of laboratory findings was confirmed by using updated microbiology guidelines such as CLSI (48).

### 2.8 Data processing and analysis

Data were edited, cleaned, and double-entered into EpiData version 3.1 and then exported to the statistical package for social science statistics version 26 for further analysis. Descriptive analyses were summarized using frequency and percentage and presented in texts, tables, and figures. Binary logistic regression was analyzed to assess associated factors with the prevalence of bacteria isolated from the hands of housemaids. The variables with a value of *p* ≤ 0.25 were fitted into the multivariable analysis. A Hosmer and Lemeshow statistical test was carried out to check the goodness of fitness. Variables were selected through a backward, stepwise selection technique. The odds ratio with a respective 95% confidence interval was used to measure the strength of the association. A *p* value of <0.05 was considered statistically significant.

### 2.9 Ethical consideration

The Institute of Health Sciences at Jimma University’s IRB granted ethical clearance for the study which was carried out under reference number IHRPGS/437/22. Every respondent was asked for their informed and oral consent. Codes were used to maintain the complete confidentiality of the information collected from study participants, including their privacy. Concerning parties, such as research participants and households, would be connected to the atypical clinical result. When gathering data, personal safety measures were taken to prevent the spread of COVID-19 from the data collector to study participants and vice versa. These measures included wearing a mask, and gloves, wiping hands with sanitizer or alcohol, and washing hands with detergent.

## 3 Results

### 3.1 Sociodemographic characteristics

**Figure 2:**
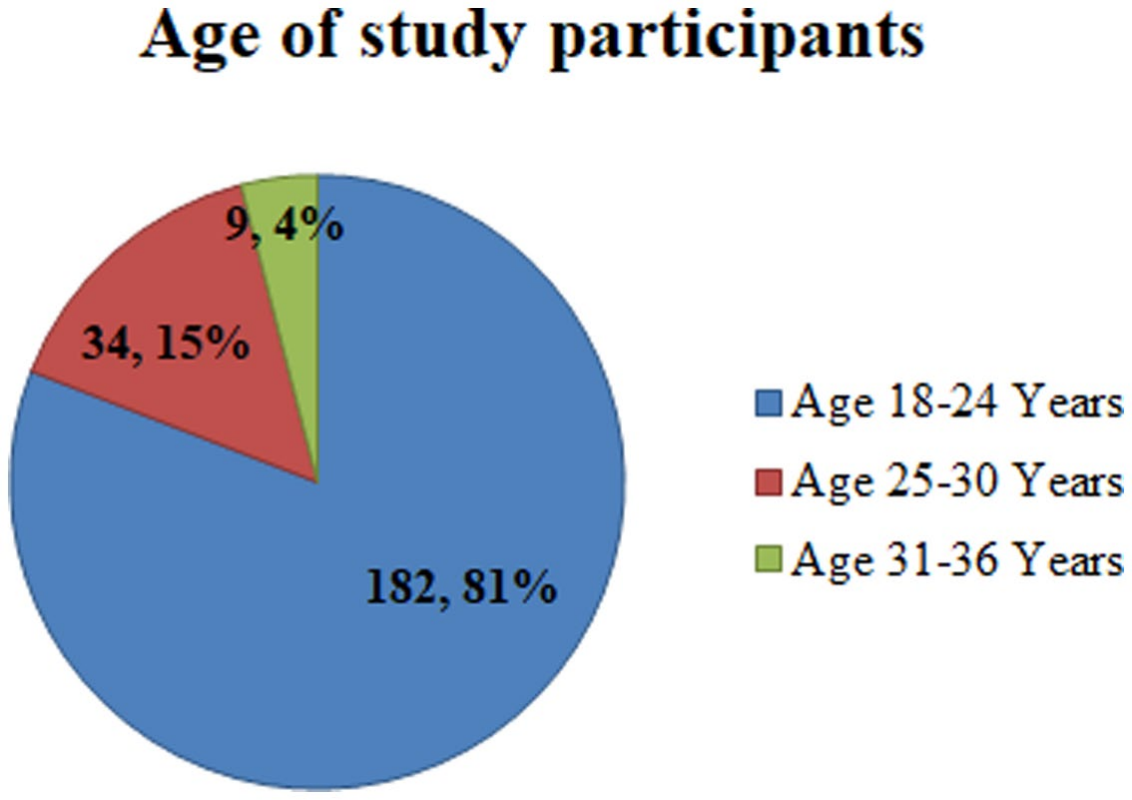
Age category of housemaids (*n* = 225) working in Jimma City, Southwest Ethiopia, 2022.

**Figure 3:**
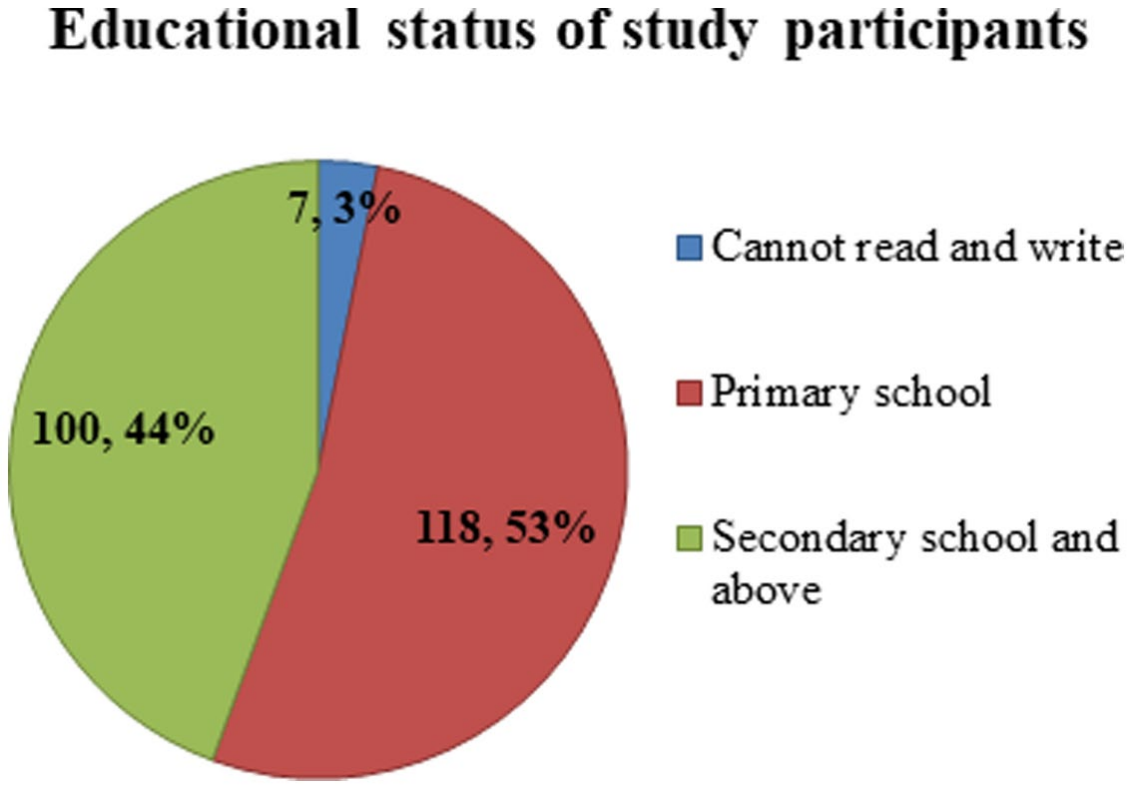
Educational status of housemaids (*n* = 225) working in Jimma City, Southwest Ethiopia, 2022.

Two hundred and twenty-five study subjects participated in this study, with a response rate of 96.2%. All the respondents were women. The age of study participants ranged from 18 to 36, with a mean age of 21.41 ± SD of (3.961). The majority, 182(81%) and 34(15%) of the study participants were between the age categories of 18–30 and 18–24 years, respectively (Figure 2). More than half (53%) of study participants attended primary school, while 7(3%) of respondents could not read or write (Figure 3).

### 3.2 Prevalence of bacterial isolates

The overall prevalence of one or more bacteria isolated from the hands of housemaids was 72% (95% CI: 66.2–77.8). The total number of bacteria isolated from hand swab samples was 224. *Staphylococcus aureus* 71(31.6%) was the predominant bacterial species, followed by *Klebsiella* species 52(23.1%) and *E. coli* 48(21.3%), whereas the least isolated bacteria was *Coagulase-negative staphylococci* (0.90%). However, bacteria were not isolated from 63(28%) of the study participants’ hands (Table 2).

**Table 2:** Types of bacterial isolates from the hands of housemaids (*n* = 225) in Jimma City, Southwest Ethiopia, 2022.

Bacteria isolates	Frequency	Percent
*Staphylococcus aureus*	71	31.6
Coagulase-negative staphylococci	2	0.9
*Escherichia coli*	48	21.3
*Salmonella* species	3	1.3
*Shigella* species	15	6.7
*Klebsiella* species	52	23.1
*Proteus* species	33	14.7

#### 3.2.1 Factors associated with bacterial isolation

In bivariable logistic regression, variables including fingernail status, frequent handwashing, how to wash hands, washing hands frequently with soap/other detergents, following five steps to wash hands the right way, removing a watch, ring, and bracelet during handwashing, and washing hands for 20 s were significantly associated with bacterial isolates from hands. In multivariable logistic analysis, all variables with a value of *p* ≤0.25 in bivariable analyses were included. Fingernail status and removing a watch, ring, and bracelet during handwashing were found to be significantly associated with bacterial isolates from the hands of housemaids, with a value of *p* < 0.05 (Table 3).

**Table 3:** Factors associated with the bacterial isolation from the hands of housemaids (*n* = 225) in Jimma City, Ethiopia, 2022.

Study variables	Category	Bacterial culture results from hands		Chi-square and *p* value	COR (95%CI)	Sig.	AOR (95% CI)
		Negative	Positive				
Age of housemaids	18–24 years	49	133	χ^2^_(df = 2)_ = 3.534	1.24 (0.571, 2.839)		
	25–30 years	9	25	*p* = 0.1710	5.02 (0.029, 1.951)		
	≥31 years	5	4		1	1	1
Educational status	Cannot read and write	1	6	χ^2^_(df = 2)_ = 1.835	9 (3.012, 12.002)		
	Primary school	30	88	*p* = 0.3990	0.84 (0.192, 11.219)		
	Secondary and above	32	68		1	1	1
Fingernail status	Not trimmed	10	39	χ^2^_(df = 1)_ = 1.791	6.54 (1.424, 9.375)	0.000	15.31 (10.372,22.595)**
	Trimmed	53	123	*p* = 0.1810	1	1	1
Frequently washing hands	No	2	16	χ^2^_(df = 1)_ = 2.768	5.65 (2.196, 14.532)		
	Yes	61	146	*p* = 0.0960	1	1	1
How to wash hands	Water only	22	8	χ^2^_(df = 1)_ = 0.133	6.96 (2.958, 16.369)		
	Water and soap	53	124	*p* = 0.7160	1	1	1
Frequent HW with soap or other detergents	No	3	2	χ^2^_(df = 1)_ = 2.216	6.00 (2.713,13.270)		
	Yes	50	122	*p* = 0.1370	1	1	1
Follow five steps to wash hands the right way	No	36	100	χ^2^_(df = 1)_ = 1.715	10.71 (2.500,5.861)		
	Yes	25	46	*p* = 0.1900	1	1	1
Removing watch, ring, and bracelet during HW	No	33	128	χ^2^_(df = 1)_ = 5.517	1.97 (0.820, 4.743)	0.008	20.844 (2.190,9.842)*
	Yes	30	34	*p* = 0.0190	1	1	1
Washing hands for 20 s	No	28	89	χ^2^_(df = 1)_ = 3.283	3.02 (1.388, 6.572)		
	Yes	35	73	*p* = 0.070	1	1	1

CI, Confidence interval; 1: for reference category; COR, Crude odd ratio; AOR, Adjusted odd ratio; HW, Handwashing. ^*^p value < 0.05. ^**^p value < 0.001.

This study suggested that housemaids who did not trim their fingernails were 15.31 times more likely to have tested positive for bacteria cultures from the hand swabs compared to housemaids who trimmed their fingernails (adjusted odd ratio = 15.31, 95% confidence interval: 10.372, 22.595). Housemaids who did not experience the removal of a watch, ring, or bracelet during handwashing had 20.844 times higher odds of positive bacteria cultures than their counterparts (adjusted odd ratio = 20.844, 95% confidence interval: 2.190, 9.842; Table 3).

### 3.3 Antimicrobial susceptibility pattern of bacteria isolates

The majority of *Staphylococcus aureus* were susceptible to chloramphenicol (*n* = 70; 98.6%) followed by 46 (64.8%) and 42 (59.2%) sensitive to gentamicin and tetracycline, respectively. *Coagulase-negative staphylococci* were sensitive to all tested antibiotics (Table 4).

**Table 4:** Antimicrobial susceptibility pattern of bacteria isolates from the hands of housemaids in Jimma City, Southwest Ethiopia, 2022.

Bacteria isolate	Total	SP	TE [*n* (%)]	CRO [*n* (%)]	C [*n* (%)]	GEN [*n* (%)]	CAZ [*n* (%)]
*Staphylococcus aureus*	71	Sensitivity	42 [59.2]	NA	70 [98.6]	46 [64.8]	NA
		Intermediate	9 [12.7]		1 [1.4]	25 [35.2]	
		Resistant	20 [28.2]		0 [0.0]	0 [0.0]	
Coagulase-negative staphylococci	*2*	Sensitivity	2		2	2	
		Intermediate	0		0	0	
		Resistant	0		0	0	
*E.coli*	48	Sensitivity	36 [75.0]	38 [79.2]	42 [87.5]	30 [62.5]	37 [77.1]
		Intermediate	0 [0.0]	4 [8.3]	0 [0.0]	18 [37.5]	5 [10.4]
		Resistant	12 [25.0]	6 [12.5]	6 [12.5]	0 [0.0]	6 [12.5]
*Salmonella* species	3	Sensitivity	1	2	3	1	2
		Intermediate	1	0	0	1	1
		Resistant	1	1	0	1	0
*Shigella* species	15	Sensitivity	3 [20.0]	8 [53.3]	12 [80.0]	12 [80.0]	8 [53.3]
		Intermediate	5 [33.3]	3 [20.0]	3 [20.0]	0 [0.0]	3 [20.0]
		Resistant	7 [46.7]	4 [26.7]	0 [0.0]	3 [20.0]	4 [26.7]
*Klebsiella* species	52	Sensitivity	25 [48.1]	28 [53.8]	46 [88.5]	31 [59.6]	28 [53.8]
		Intermediate	15 [28.8]	14 [26.9]	6 [11.5]	21 [40.4]	7 [13.5]
		Resistant	12 [23.1]	10 [19.2]	0 [0.0]	0 [0.00]	17 [32.7]
*Proteus* species	33	Sensitivity	23 [69.7]	9 [27.3]	33 [100.0]	27 [81.8]	5 [15.2]
		Intermediate	1 [3.0]	8 [24.2]	0 [0.0]	5 [15.2]	20 [60.6]
		Resistant	9 [27.3]	16 [48.5]	0 [0.0]	1 [3.0]	8 [24.2]

SP, Sensitivity pattern; TE, Tetracycline; CRO, Ceftriaxone; C, Chloramphenicol; GEN, Gentamycin; CAZ, Ceftazidime; *n*, number; %, percent; NA, not applicable.

However, approximately 20 (28.2%) of isolated *S. aureus* were resistant to tetracycline. In addition, no resistance was reported regarding *Coagulase-negative staphylococci*. Regarding *S. aureus* isolates, no resistance was reported to chloramphenicol or gentamicin (Table 4).

For *E. coli* (*n* = 48), approximately 36 (75%), 38 (79.2%), 42 (87.5%), and 37 (77.1%) isolates were sensitive to tetracycline, ceftriaxone, chloramphenicol, and ceftazidime. For *Salmonella* species, all three isolates and two of them were sensitive to chloramphenicol, ceftriaxone, and ceftazidime. Approximately 80% of the *Shigella* isolate was sensitive to chloramphenicol and gentamicin, respectively. For *Klebsiella* isolates (*n* = 52), 46 (88.5%), 31 (59.6%), and 28 (53.8%) were sensitive to chloramphenicol, gentamicin, ceftriaxone, and ceftazidime. Moreover, 100, 81.8, and 69.7% of *P*r*oteus* species were sensitive to chloramphenicol, gentamicin, and tetracycline, respectively (Table 4).

One-quarter of *E. coli* isolate was resistant to tetracycline, ceftriaxone, chloramphenicol, and ceftazidime, while a single isolate of *Salmonella* was resistant to tetracycline, ceftriaxone, and gentamicin. Most *Shigella* isolates were resistant to tetracycline (46.7%), ceftriaxone (26.7%), and ceftazidime (26.7%). Moreover, 16 (48.5%), 9 (27.3%), and 8 (24.2%) of isolated *Proteus* species were resistant to ceftriaxone, tetracycline, and ceftazidime, respectively (Table 4).

However, no resistance was reported to chloramphenicol regarding *Salmonella*, *Shigella*, *Klebsiella,* and *Proteus* species. In addition, no resistance was reported regarding gentamicin on isolates of *E. coli* and *Proteus* (Table 4).

From total bacteria isolates, no resistant chloramphenicol was recorded among six (85.7%), namely *S. aureus*, *Coagulase-negative staphylococci*, *Salmonella*, *Shigella*, *Klebsiella* species, and *Klebsiella* species Similarly, no gentamicin resistance was recorded among four (57.1%), namely *S. aureus*, *Coagulase-negative staphylococci*, *E.coli*, and *Klebsiella* species (Table 4).

## 4 Discussion

Housemaids with poor hand hygiene could be potential sources of infection due to pathogenic bacteria, which can cause food contamination and, consequently, foodborne diseases that pose a potential risk to public health (18, 25). Due to the scarcity of published information, the bacterial contamination level among housemaids in Ethiopia is underexplored. Therefore, the present study was undertaken to assess the prevalence and antibiotic susceptibility profile of bacteria isolated from the hands of housemaids in Jimma City, Ethiopia.

The prevalence of bacterial isolation from the hands of housemaids was 72%. This result is comparable with a study conducted in Tripoli, Libya with the prevalence of bacterial growth (71.41%) (51). The bacteria isolation from hands could be due to poor hand hygiene practices such as untrimmed fingernails and not removing the watch, rings, and bracelets during handwashing. Thus, jewelry could lead to bacteria colonizing the hands (52). In addition, bacterial isolation from the hands of housemaids illustrates the concept of fecal contamination (53). The other reason might be the quality of the handwashing water. Pieces of evidence revealed that bacterial contamination of hands is significantly affected by handwashing water (54, 55).

However, the result is higher than the reported prevalence by a previous study performed in Iran with a bacteria isolation rate of 62.2% (25), Egypt with a positive culture for one or more microbial contaminants (60%) (56), Sudan with a carrier of pathogenic bacteria (23.2%) (24), and in different parts of Ethiopia: Jimma (49.6, 6.9, and 19.0%) (11, 15, 20), Gondar town (13. 2%) (1), Debre Markos (29.5 and 46.7%) (16, 17), and Dessie town (59.4%) (12). On the other hand, the result is lower than the study conducted in Mauritius, in which the prevalence of bacteria growth from hands was 91.0% (26), and in Ethiopia, at the University of Gondar Referral Hospital (UoGRH), the prevalence of bacterial isolation was 83.9% (13). The observed discrepancy in the bacteria isolation rate might be due to the differences in the study settings and periods, study participants, hygiene practices, and referent pressure for hygienic conditions.

In this study, *S. aureus* was the predominant bacterial species in the hands of housemaids, with an isolation rate of 31.6%. This result is comparable to a study conducted in the ICUs of United States medical centers (30.0%) (57) and UoGRH, Ethiopia, where the prevalence of *S. aureus* was 34% (13). The isolation of *S. aureus* could be because it is a pathogenic bacterium that is part of the normal flora of the skin and other body parts.

However, the result is lower than the study performed in Sudan (71.8%) (24), Iran (46%) (25), Nigeria (68.9%) (58), Eritrea (63.1%) (59), and Ethiopia; Addis Ababa Regional Laboratory (50.0%) (60) and Ethiopia’s Debre Markos Comprehensive Specialized Hospital (DMCSH) (46.2%) (19), but higher than the study conducted in Alexandria, Egypt (22%) (56), Eastern India (3.44%) (5), and Ethiopia; University of Gondar (16%) (14); Debre Markos (5%) (17); Gondar Town (16.5%) (61), and Jimma University main campus (23.5%) (15). The discrepancy in the isolation rate of *S. aureus* might be due to differences in the sociodemographic characteristics, the study periods, the study settings and participants, the working environment, and the sample size.

The majority of *S. aureus* was sensitive to chloramphenicol (98.6%), followed by gentamycin (64.8%) and tetracycline (59.2%). This result is higher compared to the results reported in the UoGRH with sensitivity to chloramphenicol (76.9%) (13), the Addis Ababa regional laboratory with sensitivity to chloramphenicol (53.7%) (60), and DMCSH with sensitivity to chloramphenicol (66.7%) (19). Regarding resistance, isolates of *S. aureus* were resistant to tetracycline (28.2%). The result is lower than the study performed by Addis Ababa Regional Laboratory (74.30%) (60), Debre Markos (54.5%) (17), and UoGRH (64.1%) (13), but the result is higher than the study conducted at the University of Gondar (21.9%) (14). Different mechanisms play a pivotal role in how *S. aureus* became resistant to antimicrobials. The antimicrobial resistance of *S. aureus* to tetracycline might be due to increased efflux, the production of β-lactamase to β-lactam-sensitive antibiotics, the presence of acetyltransferase, a decrease in accumulation of macrolide antibiotics, the expression of the mec gene, and the formation of alternative pathways for sulphonamides (62). The reason for the antimicrobial resistance could be the dissemination of the strain (31). Moreover, the antimicrobial resistance of *S. aureus* could be determined by a lack of access to appropriate antimicrobial susceptibility tests and bacteriological diagnosis that could lead to the misuse of antimicrobials by patients (16).

In the present study, the isolation rate of *E. coli* was 21.3%, which is comparable with a study conducted in India (20.68%) (5). The result is lower than the study performed in Iran (29.2%) (51), Nigeria (25.0%) (58), and Jimma University Specialized Hospital (JUSH) (25.4%) (20). However, the result is higher compared to previous studies conducted in Mauritius (0.5%) (26), in the ICUs of United States medical centers (7.1%) (57), and in the different parts of Ethiopia: Jimma University (10.9%) (15), Gondar Town (3.1 and 1.9%) (1, 61), Debre Markos (2.7%) (17), UoGRH (5.9%) (13), University of Gondar (2.67%) (14), DMCSH (3.9%) (19), and Addis Ababa Regional Laboratory (3.6%) (60). The isolation of *E. coli* illustrates the concept of fecal contamination in the hands of housemaids. In addition, De Alwis et al. (53) revealed that contaminated surfaces such as toilets and washrooms could be the sources of contamination of the hands when a person comes into contact.

The majority of *E. coli* isolates (87.5%) were sensitive to chloramphenicol. This result is lower than a study conducted in Gondar town (100%) (1); however, the result is higher compared to other studies with a sensitivity of 50% (11, 14, 17, 60), 75% (13), (66.7%) (19) and 56% to chloramphenicol (16). Similarly, approximately 79.2 and 75% of *E. coli* isolates were sensitive to ceftriaxone and tetracycline which is higher than the results reported in previous studies (11, 16, 17, 19, 60). However, resistance to tetracycline, ceftriaxone, chloramphenicol, and ceftazidime was reported for *E. coli* isolates (12.5%). It is lower than the results reported in other studies (11, 14, 16, 17, 60). A study conducted in the JUSH showed that *E. coli* isolates were resistant to ceftriaxone (73%) and ceftazidime (65%) (20). Another study carried out in DMCSH revealed that *E. coli* was resistant to tetracycline (44.4%), chloramphenicol (22.2%), and ceftazidime (33.3) (19). *Escherichia coli* were sensitive to chloramphenicol, which might be due to decreased levels of acetyl coenzyme A incat-expressing CM2555 cells in the presence of chloramphenicol (63). The antimicrobial resistance of *E. coli* to antimicrobial drugs could be due to its outer membrane and the expression of numerous efflux pumps (64). In addition, the antimicrobial resistance of *E. coli* might be due to the transmission of resistance genes and the unrestricted use of antimicrobials that perpetuate antimicrobial-resistant plasmids (65). Furthermore, other contributors to antimicrobial resistance are the spread of *E. coli*-resistant strains, overuse or inappropriate prescribing, use of antibiotics in livestock, hygiene/fecal colonization, and antibiotic resistance mechanisms (β-lactams) (64).

In the current study, the isolation rate of *Shigella* species was 6.70%, which is comparable to the result reported in Debre Markos University, northwest Ethiopia (5.9%) (18). The isolation of *Shigella* species from the hands of housemaids illustrates poor hand hygiene practices due to fecal contamination. The other reason might be due to cross-contamination of hands with surfaces such as toilets and washrooms (53). The result is lower than a study performed in Gondar town with a prevalence of 10.1% (1), but higher compared to previous studies conducted in Jimma town (0.2%) (11), the University of Gondar (2.7%) (14), and Debre Markos Referral Hospital (5.4%) (16). The variation might be due to differences in the demographic characteristics, study settings and period, study design and sampling techniques, and geographical variation of study areas.

Concerning the antimicrobial resistance profile of isolates, approximately 80% of isolated *Shigella* species were sensitive to chloramphenicol and gentamicin, respectively. This is comparable to a study performed in Gondar town (1) and Debre Markos University (18), but the result is higher than previous studies performed at the Debre Markos Referral Hospital (16) and the University of Gondar (14). On the other hand, approximately 46.7 and 26.7% of isolated *Shigella* species were resistant to tetracycline, ceftriaxone, and ceftazidime. This is consistent with reported results in Gondar town (1) and Debre Markos University (18). The result is lower than a previous study performed in Debre Markos Referral Hospital (16), Gondar town (1), and Jimma town (11), but higher than a previous study in Gondar town (1) and Jimma town (11). The resistance of *Shigella* species to tested antibiotics might be due to high descriptions from clinics available in the locality as well as self-medication. In addition, it might be due to genetic diversity (66).

In the present study, the isolation rate of *Klebsiella* species was 23.1%, which is higher than the previous study conducted in different parts of Ethiopia: Debre Markos Referral Hospital (4.3%) (16), Gondar town (1.67%) (14), Debre Markos University (2.7%) (17), UoGRH (12.5%), Gondar town (5.5%) (61), and ICUs of US medical centers (11.8%) (57). Regarding the antimicrobial susceptibility pattern of *Klebsiella* species, the majority of *Klebsiella* isolates (88.5%) were sensitive to chloramphenicol. The result is in line with a previous study conducted in Ethiopia (17). While antibiotic resistance to tetracycline, ceftriaxone, and ceftazidime among *Klebsiella* isolates was recorded, a study performed in different parts of Ethiopia showed antibiotic resistance of *Klebsiella* species regarding chloramphenicol, tetracycline, ceftriaxone, gentamicin, and ceftazidime (13, 14, 16, 17, 20). The antimicrobial resistance of *Klebsiella* species might be due to its strains having a 𝛽-lactam ring provided with a Zwitterionic structure (20). Another aggravating factor for antimicrobial resistance of *Klebsiella* species might be the self-prescribing of antibiotics by a few people due to the availability of antibiotics on the market in the study area and inappropriate use of antibiotics (60).

The isolation of *Proteus* species is 14.7% in the present study, which is inconsistent with Debre Markos University (1.4%) (17), JUSH (2.0%) (20), Jimma University (2.2%) (15), and UoGRH (9.6%) (13). Moreover, we observed antimicrobial resistance of *Proteus* species to ceftriaxone, tetracycline, and ceftazidime in the current study. It is consistent with a previous study that showed antimicrobial resistance recorded for tested drugs such as ceftriaxone, tetracycline, and ceftazidime (13, 17, 20).

In sum, sensitivity to chloramphenicol on most bacteria isolates was observed, but resistance to tetracycline, ceftriaxone, and ceftazidime among isolates *E. coli*, *Salmonella*, *Shigella*, *Klebsiella* species, and *Proteus* species was observed in the present study. At present, antimicrobial resistance is an emerging global challenge that results in the spread of infectious diseases that could affect human populations (38, 39). This might be due to a complex set of causes such as biological processes, human behaviors, and social factors that support the microbes to multiply, carry on, and produce harm (37). In addition, antimicrobial resistance could be due to the evolutionary processes or natural phenomena to which microbes tend to adapt (36, 37). The other reason for AMR could be the inappropriate use of drugs by the community, the use of antibiotics in animals, or the external environment (18, 38, 39). Moreover, the global connection of a large human population allows microbes to move from place to place and spread, allowing them to easily enter the environment (39).

In comparison to housemaids who clipped their fingernails, housemaids who neglected to do so were 15.31 times more likely to have positive bacteria cultures (AOR = 15.31, 95% CI: 10.372, 22.595). This result is in line with the result reported in Poland (OR: 7.1; 95% CI:1.83, 27.39) (67). The result is also supported by a study performed by Mengist et al. (17). In addition to limiting the use of proper hand hygiene, long or sharp fingernails might promote bacteria development. The likelihood of positive bacterial isolation was 20.844 times higher in housemaids who did not remove their accessories before washing their hands than their counterparts (AOR = 20.844, 95% CI: 2.190, 9.842). During handwashing, accessories must be taken off to prevent the growth and spread of harmful microorganisms. They should be well rinsed unless doing so would cause bacterial growth (53).

### 4.1 Limitations

This study did not identify important microbial hand contaminants such as *V. cholerae*, *Helicobacter, and Campylobacter* due to constraints on resources. In addition, some antimicrobial susceptibility tests were performed in this study due to the lack of antimicrobial disks. Furthermore, a multidrug resistance test was not conducted for bacteria isolated from the hands of housemaids.

## 5 Conclusion

Housemaids’ hands are very important potential sources of disease-causing bacterial pathogens that would result in the potential risk of gastrointestinal tract infections. Fingernail status and the removal of accessories during handwashing were found to be significantly associated with the prevalence of bacterial isolation from the hands of housemaids. Moreover, most of the bacterial isolates were sensitive to chloramphenicol and gentamycin, while the majority of them were resistant to tetracycline, vancomycin, and ceftazidime.

Therefore, keeping fingernail status short, removing accessories during handwashing, and practicing good hand hygiene are crucial to preventing and controlling antimicrobial-resistant microbes. In addition, any community health worker, regional, national, and other stakeholders who are engaged in community health should create awareness about regular handwashing and its relevance in the prevention and reduction of pathogenic microorganisms from hands in the wider community.

## Data availability statement

The original contributions presented in the study are included in the article/supplementary material, further inquiries can be directed to the corresponding author.

## Ethics statement

The study was conducted after obtaining an ethical clearance with reference number IHRPGS/437/22 approved by the Institutional Review Board of the Institute of Health Sciences, Jimma University. The studies were conducted in accordance with the local legislation and institutional requirements. The participants provided their written informed consent to participate in this study.

## Author contributions

TA: Conceptualization, Data curation, Formal analysis, Funding acquisition, Investigation, Methodology, Project administration, Resources, Software, Supervision, Validation, Visualization, Writing – original draft, Writing – review & editing. NG: Visualization, Writing – original draft, Writing – review & editing. GZ: Visualization, Writing – original draft, Writing – review & editing. TT: Formal analysis, Validation, Visualization, Writing – original draft, Writing – review & editing. TG: Formal analysis, Validation, Visualization, Writing – original draft, Writing – review & editing.
